# A randomized controlled trial on the effects of collagen sponge and topical tranexamic acid in posterior spinal fusion surgeries

**DOI:** 10.1186/s13018-017-0672-2

**Published:** 2017-11-06

**Authors:** Derong Xu, Qianyu Zhuang, Zheng Li, Zhinan Ren, Xin Chen, Shugang Li

**Affiliations:** Department of Orthopedic Surgery, Peking Union Medical College Hospital, No.1 Shuai Fu Yuan, Wang Fu Jing Street, Beijing, 100730 China

**Keywords:** Spinal fusion surgery, Collagen sponge, Topical TXA, Blood loss

## Abstract

**Background:**

This is a randomized controlled trial research to assess the hemostatic efficacy of gelatin sponge, collagen sponge, and topical use of tranexamic acid (TXA) on postoperative blood loss in posterior spinal fusion surgeries.

**Methods:**

We recruited patients with spinal degenerative diseases into the study from November 2013 to October 2016. All the participants were assigned to 3 groups using a simple, equal-probability randomization scheme: group A is a control group utilizing gelatin sponge, while groups B and C are experimental groups, applying collagen hemostatic sponge and topical TXA respectively. Postoperative blood loss, rates of transfusion, and hospitalization were compared among the 3 groups.

**Results:**

In our study, the volume of drainage and blood content in drainage on the first postoperative day (POD 1) of patients in the experimental groups were significantly less than those in the control group, as well as rates of transfusion and postoperative hospitalization (*P* < 0.05). When compared with the control group, the volume of drainage decreased by 22.7% in group B and 56.2% in group C, while the blood content in drainage decreased by 28.8 and 75% respectively.

**Conclusions:**

In this study, collagen and topical use of TXA have both proven to be effective and safe for patients undergoing posterior spinal fusion surgeries, while TXA has exhibited better efficacy. The total amount of perioperative blood loss reduced significantly without increasing incidence of related complications.

**Trial registration:**

A randomized controlled trial for effects of collagen sponge and topical tranexamic acid in posterior lumbar fusion surgeries. ChiCTR-IIR-17010785.

## Background

Effective measures to control perioperative bleedings is a common issue that should be taken seriously, especially in complex and high-risk multilevel spinal fusion surgeries. Excessive blood loss may result in diverse undesirable consequences, such as severe anemia, massive transfusions, prolonged hospitalization, increased incidence of wound infections, and medical expenses [1, 2]. Therefore, many blood protection measures have been implemented to control bleeding in spinal surgeries, including hypotensive anesthesia, intra-operative cell salvage systems, and application of hemostatic agents [3].

The gelatin sponge has been used in surgical procedures for several decades; it has no bioactivity and controls bleeding mainly by volume expansion and mechanical compression. In contrast to gelatin sponge, collagen sponge contains hemostatic agents that are purified from original type I collagen. So besides physical compression, the biological components in collagen sponge possess hemostatic function by activating the platelets and intrinsic coagulation pathway. In addition, Lan [4] pointed out that the excellent hemostatic effect of collagen sponge may lead to a higher platelet ratio resulting from the blood adsorption.

As an antifibrinolytic agent, tranexamic acid (TXA) can block the interaction of plasminogen and plasmin by competing with the lysine residues on the surface of fibrin to inhibit the fibrinolysis and consequently stabilize clot [5]. It could be applied intravenously or topically [6]. Lots of researches have demonstrated that intravenous TXA (IV TXA) can reduce blood loss and transfusion requirements in total knee arthroplasty (TKA), total hip arthroplasty (THA), and spinal fusion surgeries [7–9]. However, IV TXA might be accompanied with serious side effects, which are quite rare but do exist, especially in patients with hypercoagulability, severe ischemic heart diseases, and renal failure [10]. On the other hand, there are relatively fewer reports about the safety and efficacy of topical use of TXA in spinal surgeries.

The goal of this study was to evaluate the efficacy of three different methods—gelatin sponge, collagen sponge, and topical TXA in reducing blood loss in patients undergoing posterior spinal fusion surgeries. Additionally, we observed the incidence of perioperative complications, rates of transfusion, and hospitalization as well.

## Methods

This is a randomized controlled clinical study. Patients diagnosed with spinal degenerative diseases at Peking Union Medical Hospital were recruited into research from November 2013 to October 2016. The inclusion criteria were spinal degenerative diseases, such as spinal stenosis, disc diseases, and instability (e.g., grade I–II spondylolisthesis, spondylolisthesis/spondylolysis) indicated for surgical treatments. The exclusion criteria were as follows: (1) patients with comorbid severe medical diseases such as osteoporosis, anemia, renal failure, and cardiovascular diseases; (2) patients with abnormal coagulation function; (3) patients who have taken anti-platelet aggregates such as aspirin or anticoagulants in the last month; and (4) patients who had a history of thromboembolisms.

**Fig. 1:**
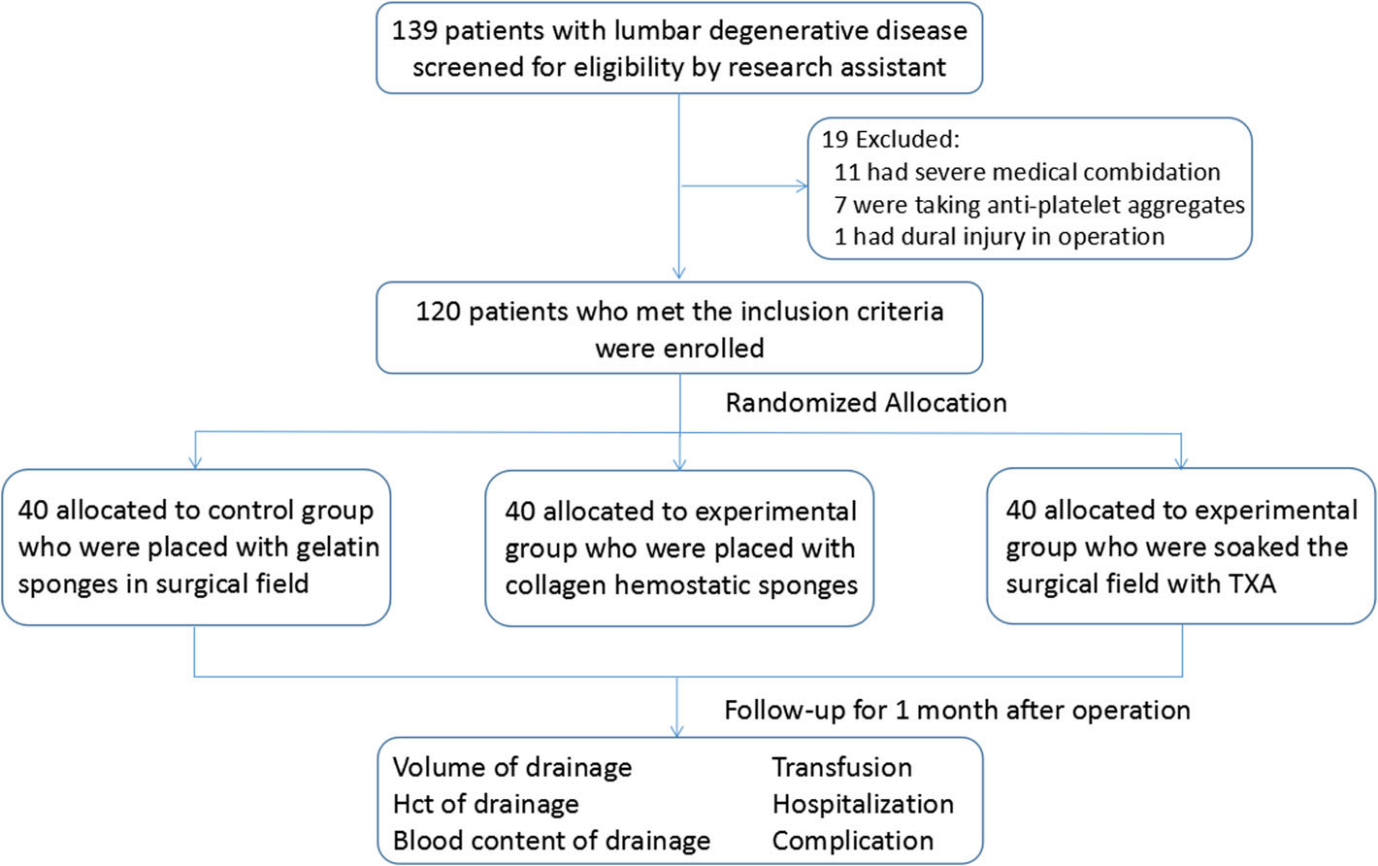
Flow chart

All the participants were assigned to 3 groups using a simple, equal-probability randomization scheme: because of limited hemostatic effect as explained before, patients utilizing gelatin sponge in group A were referred as control group, patients in group B were applied with collagen hemostatic sponge group, and patients in group C were applied with topical TXA. The participants were presented in a flow diagram in Fig. 1.

All the surgeries were performed by the same surgeons. After general anesthesia, patients were performed total laminectomy with pedicle screw instrumentation by free-hand technique. In addition to posterior bony structure decompression, patients underwent discectomy if diagnosed with disc herniation. After articular process fusion with bone grafting, we took different hemostatic measures according to group allocation. For patients in group A and group B, we applied gelatin sponges and collagen hemostatic sponges separately. According to the size of exposed spinal dura, we cut the hemostatic materials into proper shape to ensure the entire dura was covered. For patients in group C, we soaked the surgical field with TXA (1 g in 100 ml saline solution) for 5 min and then aspirated the TXA solution before stitching the wound. All the operations were accomplished by the same surgeons.

We estimated intraoperative blood loss based on weight of soaked surgical sponges and volume in suction canisters subtracting irrigation fluid added to the surgical field. At the end of the operation, we placed deep drainage below the fascia. The amount of drainage on postoperative day 1, postoperative day 2, and the total drainage volume were recorded.

The drainage was routinely removed when the drain output was less than 50 ml per 24 h. Recorded clinical data includes age, height, weight, body mass index (BMI), operative durations, surgical levels, intraoperative blood loss, related complications, and length of hospital stays.

### Transfusion

No patients received CellSaver autologous blood transfusions during operation in our study. Routine blood tests including hematocrit (HCT), hemoglobin (HGB), and coagulation index were examined on the preoperative day and at 8, 24, 48, and 72 h post-operation. Transfusion was carried out for patients with hemoglobin level less than 8 g/dL and for symptomatic patients with hemoglobin level between 8 and 10 g/dL, such as persistent tachycardia (heart rate N100 for at least 4 h), chest pain, dyspnea, and hypotension (a drop in blood pressure N20 mmHg).

### Complications

Venous Doppler ultrasonography was performed before patients discharge, and 1 month after operation, complications such as deep venous thrombosis (DVT)/pulmonary embolism (PE), spinal hematomas/seromas, and wound infections were investigated.

### Statistics

The Pearson chi-square test was used to analyze categorical variables. The differences in perioperative data among 3 groups were analyzed using the one-way ANOVA. In all analyses, the level of statistical significance was set at *P* < 0.05. All data analyses were performed with the SPSS 19.0 software package. Four parameters were compared within 3 groups: (1) volume of drainage in postoperative day (POD) 1, POD 2, and patient’s total drain output; (2) HCT of drainage in POD 1, POD 2, and POD 3; (3) blood content in drainage at different time points; and (4) transfusion rates and hospitalization durations.

This RCT study was approved by the ethical committee at Peking Union Medical College Hospital; the reference number is ZS-1000. All participants provided written informed consents for the study and surgery.

## Results

### Baseline characteristics

One hundred twenty patients who met the inclusion criteria participated in this study. According to the randomization scheme, there were 40 patients assigned to each group. The basic information of patients in these 3 groups is listed in Table 1. No significant differences in sex, age, BMI, preoperative HGB, surgical level, intra-operative blood loss, or operative time were detected in 3 groups (*P* > 0.05).

**Table 1:** Demographic date

Variable	Group A	Group B	Group C	*P*
*N*	40	40	40	
Age (year)	57.4 ± 10.7	58 ± 12.3	53.1 ± 12	*P* > 0.05
Sex				
Males	13	18	19	*P* > 0.05
Females	27	22	21	*P* > 0.05
BMI (kg/cm^2^)	24.9 ± 3.9	25 ± 3.3	25.6 ± 2.8	*P* > 0.05
Preoperative HGB (g/l)	126.8 ± 8.6	123.4 ± 10.3	139.4 ± 13.6	*P* > 0.05
Surgical level	2.31 ± 0.09	2.76 ± 0.12	2.40 ± 0.10	*P* > 0.05
Operative time (min)	144.5 ± 40.2	128 ± 40.9	121 ± 19.1	*P* > 0.05
Intra-operative blood loss (ml)	190 ± 123.2	223.8 ± 163	176 ± 100.5	*P* > 0.05
Mean duration of hospital stay (days)	7.82 ± 1.2	6.09 ± 1.3	6.13 ± 1.3	*P* = 0.001
Number of transfusion	12	3	3	*P* = 0.001

### Drainage

Our results showed that postoperative drainages were significantly different among the 3 groups (Table 2). Drainages in experimental groups were less than control group. In addition, patients with topical TXA in group C exhibited the least volume on POD 1 and of total postoperative drainage (*P* < 0.05).

**Table 2:** The information of postoperative drainage

Variable	Group A	Group B	Group C	*P*
Volume of drainage in POD 1 (ml)	232.8 ± 75.9	175.6 ± 76.8	90.9 ± 49.8	*P* = 0.001
Volume of drainage in POD 2 (ml)	74.1 ± 32.8	57.3 ± 34.5	41.1 ± 23	*P* = 0.001
Total volume of drainage (ml)	301.3 ± 110.9	232.8 ± 98	131.9 ± 78	*P* = 0.001

Most of previous studies treated volume of drainage as postoperative blood loss; however, the volume of drainage is not equal to the postoperative blood loss according to the Gross formula. Because the component of drainage varied with time varying, so postoperative blood loss does not depend on the amount of fluid loss but the pure blood contained in drainage [11, 12]. In our study, we also examined complete blood count (CBC) for every drainage sample to obtain data of HCT and hemoglobin (HGB), which were used to calculate the precise blood contained in drainage. The pure blood loss in drainage = volume of drainage × HCT/HCT_average_(HCT_average_ = HCT_pre_ + HCT_post_).

**Fig. 2:**
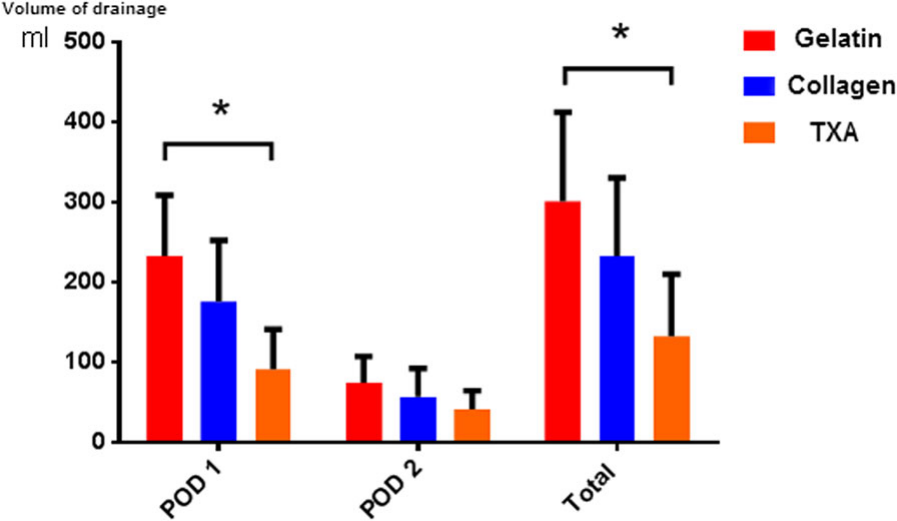
Postoperative drainage in 3 groups. The postoperative drainage in experimental groups is much less than control group in POD 1 as well as total volume, and TXA group shows the best effect. There was no significant difference in 3 groups in POD 2

**Fig. 3:**
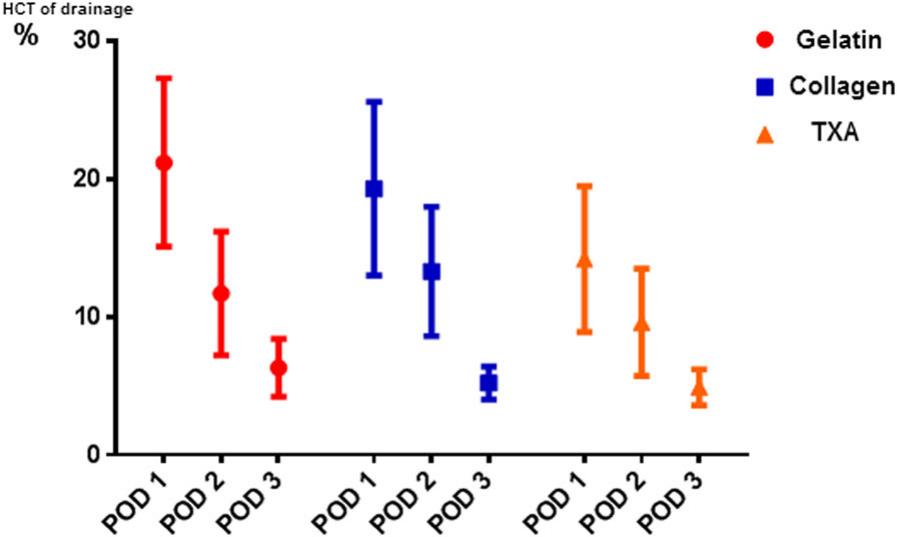
Time variation of HCT of drainage. The HCT of drainage declined gradually over time

According to the results detailed in Table 3, the average HCT of drainage in group C was lower than the other two groups (group A > group B > group C) in the postoperative 24 h; the difference showed statistical significance. While in the next 24 h, the HCT of drainage declined and no noticeable difference was observed in among groups. With the volume and HCT of drainage every day, we got accurate postoperative blood loss that is showed in Table 3 and Figs. 2 and 3. It is clear that total amount of postoperative blood loss in experimental groups was less than control group (group A > group B > group C), especially on the first day (*P* < 0.05).

**Table 3:** The actual blood contain of postoperative drainage

Variable	Group A	Group B	Group C	*P*
HCT of drainage in POD 1 (%)	21.2 ± 6.1	19.3 ± 6.3	14.2 ± 5.3	*P* = 0.001
HCT of drainage in POD 2 (%)	11.7 ± 4.5	13.3 ± 4.7	9.6 ± 3.9	*P* > 0.05
HCT of drainage in POD 3 (%)	6.3 ± 2.1	5.2 ± 1.2	4.9 ± 1.3	*P* > 0.05
Drainage blood in POD 1 (ml)	137.1 ± 68.6	94.1 ± 52.2	34.8 ± 25.4	*P* = 0.001
Drainage blood in POD 2 (ml)	23 ± 18	20.6 ± 16.5	15.2 ± 4.5	*P* > 0.05
Total volume of drainage blood (ml)	160.2 ± 82.8	114.4 ± 60.7	39.9 ± 28.5	*P* = 0.001

### Transfusion and hospitalization

There were 12 cases (30%) received transfusion in group A, which were in contrast to 3 cases (7.5%) in group B and 3 cases (7.5%) in group C. The hospitalization in experimental groups was less than that in control group as well (7.82 ± 1.2 vs 6.09 ± 1.3 vs 6.13 ± 1.3); these two indices have significant difference between experimental groups and control group (*P* = 0.001).

### Complication

There were no perioperative complications, such as DVT/PE, postoperative hematomas or seromas, and postoperative infections in the 3 groups.

## Discussion

**Fig. 4:**
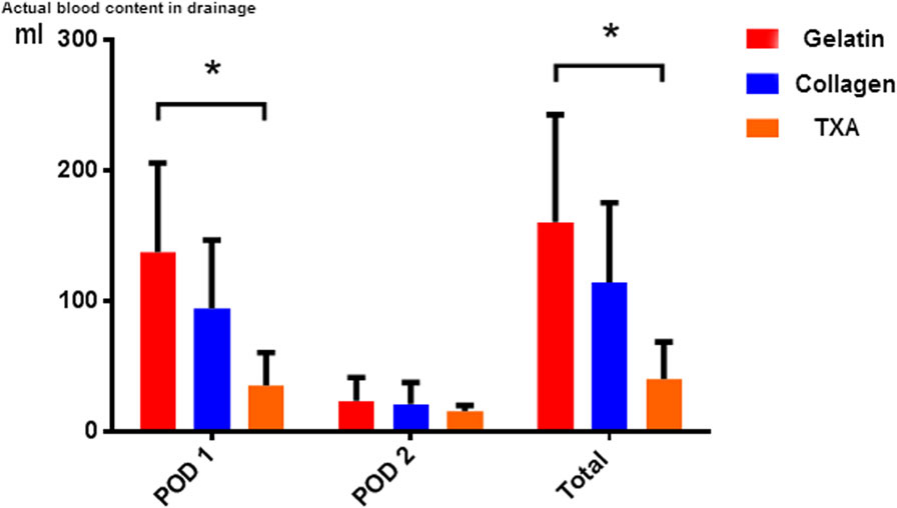
Postoperative blood content in drainage. The calculated actual postoperative blood loss in experimental groups is also less than control group in POD 1 as well as total volume, and TXA group shows the best effect. There was no significant difference in 3 groups in POD 2

Gelatin sponge, collagen sponge, and TXA have been introduced to reduce surgical bleedings as hemostatic agents for many years. In our study, the intraoperative blood loss in 3 groups had no statistical difference because the intervention happened at the end of surgery. However, the postoperative blood loss in experimental groups was much less than control group via measurement of drainage and its blood content (Fig. 4). The drainage decreased by 22.7 and 56.2% in group B and group C when compared with control group, respectively. The result is similar to many previous studies which have proven that collagen hemostatic sponge and TXA can effectively reduce postoperative blood loss in orthopedic operations [13–15]. Cho [13]soaked the absorbable gelatin sponge in thrombin and applied over the exposed spine before wound closure; the result demonstrated that patients’ postoperative drain output (93 vs 204 ml, *P* < 0.001) and consequent hospital stays (1.3 days vs 2.2 days) could be further reduced. A meta-analysis of randomized controlled trials performed by Shangquan Wang has revealed that both topical TXA and intravenous TXA have been effective in reducing blood loss and transfusion rates in patients who underwent TKA [10]. However, the used methods were completely different and there remains no consensus regarding to the relative efficacy of these two treatments. The aim of our study was to compare hemostatic effects of collagen sponge and topical TXA in a prospective randomized clinical trial for patients who undergo spinal fusion surgeries.

As mentioned before, it is more accurate to take blood content as an indicator of postoperative blood loss. According to our calculation, the blood content in drainage decreased respectively by 28.8 and 75% in collagen hemostatic sponge group and TXA group compared with gelatin sponge group. Based on results of drainage and blood content, TXA showed more effective hemostasis than collagen sponge. The reasons for this difference require further exploration, and it might relate to the fact that soaked TXA has a larger influential area than collagen’s partial coverage.

Additionally, the rate of transfusion is an important indicator to evaluate efficacy of blood conservations. Hossein Elgafy’ systematic review shows that for adult spine fusion surgery patients, the mean blood loss ranged from 650 to 2839 ml per patient and the proportion requiring transfusion ranged from 50 to 81% without plotting any strategy to reduce hemorrhage [1]. Jian Wu [16] reported that application of absorbable gelatin sponge in multilevel posterior spinal fusion surgery can decrease allogeneic blood transfusion rates (34.1 vs 58.5%, *P* = 00.046). As for TXA, Shi performed a prospective, randomized, double-blind, placebo-controlled study, in which the eligible patients were randomized to receive either a bolus dose of 30 mg/kg intravenous TXA, a maintenance dosage of 2 mg/kg/h TXA, or an equivalent volume of normal saline. The result showed that the blood transfusion rates did not vary significantly [17]. In our study, the amount of allogeneic blood transfusion of experimental groups was only 1/4 of the control group; meanwhile, there were no differences between collagen sponge group and topical TXA group. Compared with Shi’s study, topical TXA showed better effect than intravenous TXA in reducing postoperative blood transfusion.

The decreased perioperative blood loss and transfusion rate contribute to not only a lower risks of anemia and infections but also better recovery and shorter hospitalization. In our study, there was an obvious shortened time for postoperative hospitalization in experimental groups. The average postoperative stay time of collagen sponge group was 6.09 days and topical TXA group was 6.13 days, which were much shorter than 7.82 days in control group. Reasons for the improvement are probably multifactorial, including less postoperative bleeding in wound, lower incidence of anemia, and better spirits and condition that all lead to earlier functional exercises.

Furthermore, each method has its own advantages and proper scope. It is convenient for collagen hemostasis sponge to cover hemorrhagic sites whenever needed during the operation. Especially for emergency bleeding, collagen sponge also has compression function which is more effective for bleeding resulted from large vessels lacerated. For TXA, because of its liquid characteristics, it can be used for particular spinal anatomical structures and is more effective for capillary hemorrhage.

Some limitations in our study must be pointed out. The sample size was small as only 40 cases were included in each group. As many cases with severe contraindications had been excluded from our trial, it is not enough to declare that all the hemostatic measures were safe under all clinical circumstances. In the future, we will perform a further validation on a larger sample size.

## Conclusion

In this study, collagen sponge and topical TXA for patients undergoing spinal fusion surgeries were found to be effective, both of them can significantly decrease the total amount of postoperative blood loss, rates of allogeneic transfusion and hospital stays. In the meanwhile, no difference in the complication rates has been found. In comparison with collagen sponge, topical TXA is more effective in reducing postoperative drainage and bleeding, while no significant difference in transfusion rates and postoperative hospitalization.

## Abbreviations

BMI: Body mass index
DVT: Deep venous thrombosis
HCT: Hematocrit
HGB: Hemoglobin
IV TXA: Intravenous TXA
PE: Pulmonary embolism
POD 1: Postoperative day 1
POD 2: Postoperative day 2
POD 3: Postoperative day 3
THA: Total hip arthroplasty
TKA: Total knee arthroplasty
TXA: Tranexamic acid
